# Sequence Dependent Repair of 1,*N*^6^-Ethenoadenine by DNA Repair Enzymes ALKBH2, ALKBH3, and AlkB

**DOI:** 10.3390/molecules26175285

**Published:** 2021-08-31

**Authors:** Rui Qi, Ke Bian, Fangyi Chen, Qi Tang, Xianhao Zhou, Deyu Li

**Affiliations:** Department of Biomedical and Pharmaceutical Sciences, College of Pharmacy, University of Rhode Island, Kingston, RI 02881, USA; rui_qi@uri.edu (R.Q.); kebian@mit.edu (K.B.); chenfangyi@xmu.edu.cn (F.C.); qitang@uri.edu (Q.T.); xianhao_zhou@uri.edu (X.Z.)

**Keywords:** mutational spectra, mutational signatures, DNA repair, εA, AlkB, ALKBH2/3

## Abstract

Mutation patterns of DNA adducts, such as mutational spectra and signatures, are useful tools for diagnostic and prognostic purposes. Mutational spectra of carcinogens derive from three sources: adduct formation, replication bypass, and repair. Here, we consider the repair aspect of 1,*N*^6^-ethenoadenine (εA) by the 2-oxoglutarate/Fe(II)-dependent AlkB family enzymes. Specifically, we investigated εA repair across 16 possible sequence contexts (5′/3′ flanking base to εA varied as G/A/T/C). The results revealed that repair efficiency is altered according to sequence, enzyme, and strand context (ss- versus ds-DNA). The methods can be used to study other aspects of mutational spectra or other pathways of repair.

## 1. Introduction

The human genome is constantly challenged by endogenous and exogenous sources, such as reactive oxygen species (ROS), ultraviolet (UV) light, and various carcinogens [1]. These DNA damaging agents form adducts and generate unique mutational patterns, which hold promise for cancer diagnosis and prevention [2,3,4]. Researchers have developed two primary approaches to obtain these mutational features that capitalize on advances in large-scale sequencing technologies: a top-down approach referred to as *mutational signatures* and a bottom-up approach referred to as *mutational spectra* [5]. The top-down approach sequences disease-related clinical or animal samples, assembles the sequencing data into a dataset, and matches the set against healthy controls, thus generating profiles of a certain disease (e.g., cancer mutational signatures). In this way, unique mutations for distinct clinical outcomes are correlated to specific mutational signatures [4,6,7]. The top-down approach is convenient and can be performed on a large scale. However, the approach sometimes can be complicated due to confounding factors including aging, life style, inflammation, etc., which may result in an overlap of multiple layers of mutational patterns [4].

On the other hand, the bottom-up approach builds a final mutational spectra by considering the contributions of individual factors from specific chemical reactions with DNA [4]. There are several ways to obtain mutational spectra; one of them is to replicate a certain DNA adduct built in a site-specific modified genome *in vitro* or in living cells. This bottom-up approach can be more laborious due to the consideration of multiple adducts and various cellular conditions; however, the results are not sensitive to false positives. In addition, the bottom-up approach permits investigation of specific aspects of the mutational process. In terms of the mechanism of mutational process, the bottom-up method characterizes the whole journey of a DNA adduct from three aspects: formation, repair, and replication bypass of the lesion [2]. Adduct formation indicates the reactivity between the nucleophilic DNA bases and the electrophilic damaging agent. It is then possible to obtain the mutational spectra of a certain carcinogen or adduct by assembling the mutational data that are individually generated from studies of one of the three aspects. Specifically, the point mutation types are defined as the six pyrimidine substitutions (C > A, C > G, C > T, T > A, T > C, and T > G) [7]. Each of the substitutions can then be further tested by incorporating information on flanking bases (G/A/T/C) at both the 5′ and 3′ ends, which can lead to 96 possible mutation types (6 types of pyrimidine substitutions × 4 types of 5′ base × 4 types of 3′ base) [7]. The mutational patterns obtained in this manner provide unique mutagenic properties for a certain adduct/carcinogen.

Here, we used the bottom-up mutational spectra analysis to investigate the repair aspect of an individual DNA adduct, 1,*N*^6^-ethenoadenine (εA), by the AlkB family repair enzymes. Specifically, we chemically synthesized εA into oligonucleotides with all of the combinations of the 5′ and 3′ flanking bases (16 sequence contexts in total) and studied its repair efficiency by *E coli* AlkB and its human homologs ALKBH2 and ALKBH3. The reasons for choosing εA as the target were three-fold: first, εA is an important endogenous biomarker for lipid peroxidation (LPO), which is initiated by harmful reactive oxygen species (ROS) generated under inflammation or disease conditions [8,9]. Second, εA is generated by exogenous carcinogens such as vinyl chloride (VC) and chloroacetaldehyde (CAA) [10,11]. Third, researchers have studied εA repair by AlkB and ALKBH2 and 3 under different sequences and strand contexts (single-strand (ss)- versus double-strand (ds)-DNA) [12,13]. However, the results are not easy to compare without a systematic study including enzyme, sequence, and strand conditions. The three AlkB family proteins belong to the Fe(II)/2-oxoglutarate (2OG)-dependent dioxygenases, which have been reported to oxidatively de-alkylate various DNA adducts including εA, 1-methyladenine, and 3-methylcytosine by direct reversal repair [14]. Results demonstrate that εA repair by the AlkB family enzymes is sequence dependent, enzyme specific, and strand dependent. Additionally, the methods reported here show the promise of using mutational spectra analyses to (1) investigate different aspects of the mutational processes of DNA lesions and (2) to construct mutational spectra of DNA damaging agents in a stepwise manner.

## 2. Synthesis of Oligonucleotides Containing εA and Purification of Proteins

To compare the efficiencies of εA repair under different sequence contexts, we designed a sequence (5′-GACCNXNGCC-3′, X = εA, N = G/A/T/C) that allows the εA lesion to exist under all 16 possible combinations of the 5′ and 3′ neighboring bases. Each of the sixteen 10mer oligonucleotides were chemically synthesized by applying automated solid-phase phosphoramidite chemistry, purified by HPLC and characterized by high resolution mass spectrometry (see the Experimental Procedures section in Supporting Information for detailed information) [15]. Genes for the expression of the AlkB protein and its human homologs, ALKBH2 and ALKBH3, were cloned into a pET-28a(+) vector [15]. The three proteins were then expressed in *E. coli* cells, which were isolated and purified by affinity chromatography as described in the Experimental Procedures section in Supporting Information.

## 3. Repair of εA in Different Sequence Contexts by the AlkB Family Enzymes

**Figure 1:**
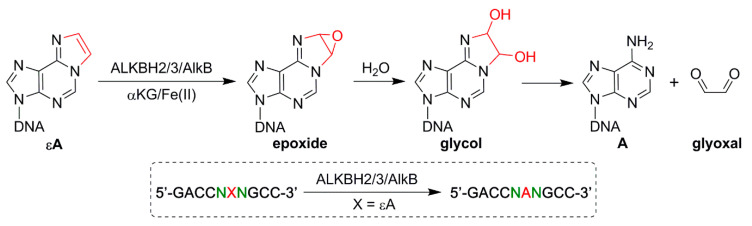
Mechanism of the AlkB family DNA repair enzymes dealkylating εA and the design of the 16 sequence contexts for εA repair. X represents the εA adduct and N represents the G/A/T/C flanking base from the 5′ and 3′ ends.

ALKBH2, ALKBH3, and AlkB were utilized to perform εA repair reactions in both ss- and ds-DNA. AlkB and ALKBH2 have been reported to efficiently repair DNA lesions in both ss- and ds-DNA, and ALKBH3 has been demonstrated to show a preference for ss-DNA (Figure 1), so these five ss- or ds-conditions for the three enzymes were chosen for the repair reactions [12,13,14,16]. The repair efficiency of the reactions was analyzed by high resolution LC-MS. The purpose of this research was to compare the repair efficiency of εA by a certain enzyme under different sequence contexts. If the enzyme concentrations were too high and led the repair of εA in two or more sequence contexts to reach 100%, there is no way to distinguish the difference in repair efficiency in those sequence contexts. Therefore, we aimed to obtain the repair ratio of each enzymatic reaction ≤ 99% by adjusting the concentration of an individual enzyme with a fixed concentration of oligonucleotide.

## 4. Results from High Resolution LC-MS Analysis on εA Reactions

Figure S1a,b provide an LC-MS analysis example of εA repair in ss-DNA by ALKBH2 in sequence TXG (named by the sequence with flanking bases on the 5′ and 3′ end). In this example, the molecular weight of the starting material 10mer TXG is calculated to be 3035.5486 Da; its monoisotopic peak (all ^1^H, ^12^C, ^14^N, ^16^O, etc.) in the −2 charge state has a calculated mass/charge (*m*/*z*) ratio of 1516.7665. Experimentally, we observed a peak at an *m*/*z* of 1516.7634 (Figure S1a), which had an error of 2.04 ppm from the calculated *m*/*z* and demonstrated the high accuracy of the MS analysis. Following the repair reaction, most of the εA adduct was converted to adenine (Figure S1b), which is observed at an *m*/*z* of 1504.7635 (calculated *m*/*z* of 1504.7665 and 1.99 ppm error). For other starting materials and product oligonucleotide species, we observed *m*/*z* values with high accuracy as well. The area under the peak envelopes of the starting material and product was used to quantify the conversion of each reaction [16].

## 5. Sequence-/Enzyme-/Strand Context-Dependent Repair of εA

**Figure 2:**
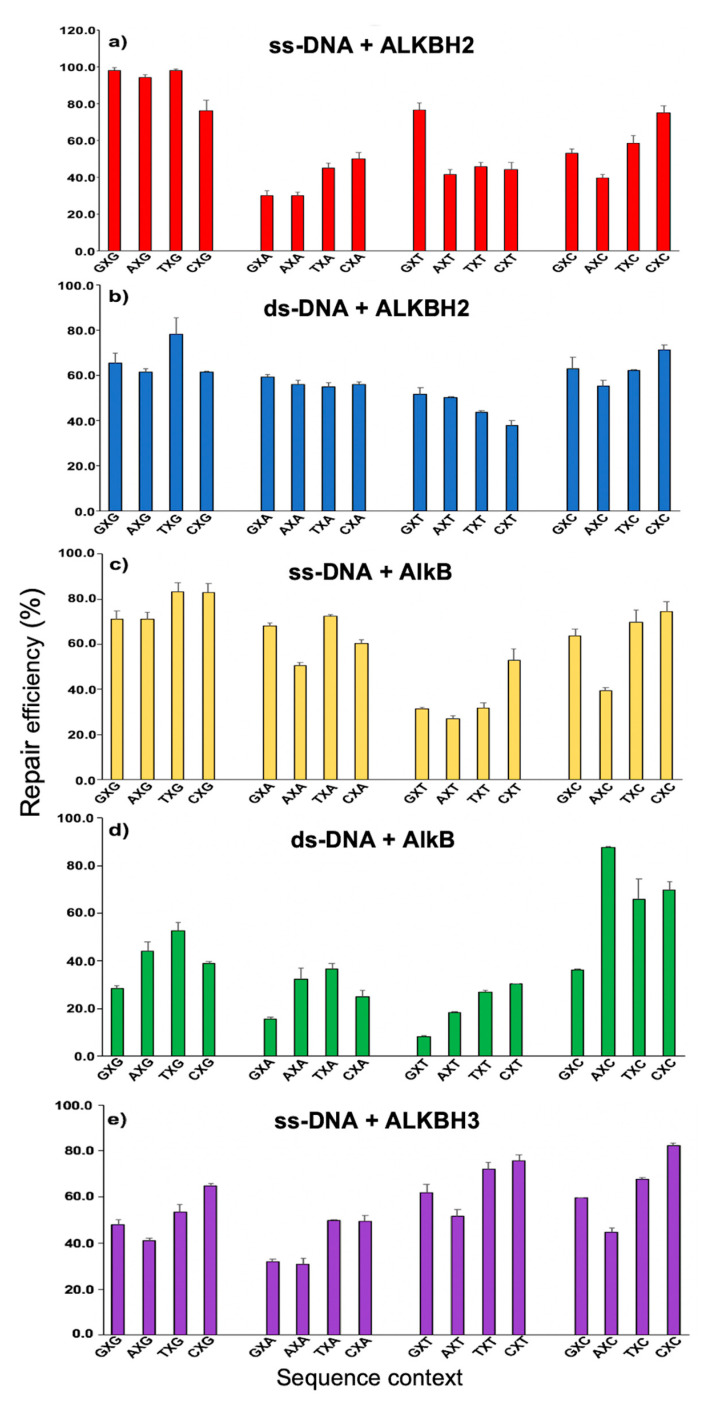
εA repair efficiencies under 16 different sequence contexts (3′ base fixed). The oligonucleotide concentration was fixed at 2.5μM. Distinct conditions are shown as follows: (**a**) ss-DNA + 2.2 μM ALKBH2 (red); (**b**) ds-DNA + 1.1 μM ALKBH2 (blue); (**c**) ss-DNA + 0.4 μM AlkB (yellow); (**d**) ds-DNA + 1.1 μM AlkB (green); and (**e**) ss-DNA + 4.0 μM ALKBH3 (purple). Percentages of repair for all reactions are summarized in Table S1. Error bars represent the standard deviation from triplicate experiments.

The repair reactions of εA were conducted under 16 sequence contexts (5′-GACCNXNGCC-3′, X = εA, N = G/A/T/C). To clearly demonstrate the different repair efficiencies, we generated a plot with the 3′ base to εA fixed and the 5′ base altered in order of the molecular weight of the 4 DNA nucleobases (G > A > T > C) (Figure 2). ALKBH2 repair of εA in ss-DNA flanked by G at the 3′ end (from 76.0% to 98.1%, Figure 2a and Table S1) was greater than A/T/C at the 3′ flanking base. Likewise, AlkB repair of ssDNA with G at the 3′ end demonstrated higher repair efficiencies than other combinations (from 70.9% to 83.0%, Figure 2c and Table S1). Among the five repair efficiency panels, three shared a similarity such that εA repair with T at the 3′ end all revealed the lowest repair efficiencies (ds-repair with ALKBH2 and ss- and ds-repair with AlkB; Figure 2b–d, respectively). Intriguingly, ALKBH3 repair of εA in ss-DNA demonstrated that εA repair with pyrimidine nucleotides (T/C) at either the 5′ or 3′ end had higher repair efficiencies than those flanked by purine nucleotides (G/A, Figure 2e). These results (Figure 2e) indicate that ALKBH3 repair of εA is likely to be size-dependent on the neighboring bases. The above observations are also demonstrated in Figure S2, which displays the repair efficiency under sequences with the 5′ end fixed and the 3′ end altered in the order G/A/T/C.

When comparing the repair efficiencies across all five conditions, ALKBH2 repair of ds-DNA was found to have the smallest repair range (40.0% difference, 2.1-fold) between 37.9% (CXT) and 77.9% (TXG), whereas AlkB repair of ds-DNA showed the largest difference (79.1%, 10.5-fold), between 8.3% (GXT) and 87.4% (AXC) (Table S1). Collectively, AlkB and its human homologs repair εA containing substrates in a sequence-dependent manner. Some previous studies have also revealed the sequence-dependent repair of different DNA adducts by various enzymes. For example, Lingaraju et al. found that εA was slightly less recognized and was removed by 3-methyladenine DNA glycosylase (Mag) within the A:T and G:C duplex tracts than in random sequences. In contrast, the excision of hypoxanthine by Mag is more efficient (by up to 7-fold) in A:T and G:C duplex contexts than in random sequences [17].

It is also worth noting that the repair efficiency for the same enzyme varies significantly under ss-DNA and ds-DNA contexts. For example, TXT is repaired by 45.7% in ss-DNA with 2.2 μM ALKBH2, but similar repair efficiencies (43.6%) were achieved in ds-DNA using only 1.1 μM ALKBH2 (Table S1). As such, εA is more readily repaired by ALKBH2 in ds-DNA than ss-DNA. In contrast, εA is less repaired by AlkB in ds-DNA than in ss-DNA. For ALKBH3, efficient repair was only observed under ss-DNA conditions (Table S1). Indeed, we could not detect any quantifiable repair product under ds-DNA conditions, even with high ALKBH3 loading. These observations are consistent with the strand preference of those enzymes, which has been reported previously [14,16]. Previously, Mishina et al. demonstrated the ability of *E. coli* AlkB and human ALKBH3 to reverse εA in 3mer and 11mer single-stranded DNA [12]. Additionally, Zdżalik et al. revealed that all three ε-adducts (εA, 3,*N*^4^-ethenocytosine (εC) and 1,*N*^2^-ethenoguanine) are repaired by ALKBH2 in ds-DNA, whilst only εA and εC in ss-DNA have low repair efficiencies; ALKBH3 only removes εC from ss-DNA [13]. Additionally, our results showed that different amounts of enzymes were needed to achieve similar repair efficiencies in ss-DNA reactions: 2.2 μM ALKBH2, 0.4 μM AlkB, and 4.0 μM ALKBH3. These findings indicate that different enzymes may have different binding affinities to εA or may provide different repair capacities to the same substrate.

In this report, we focused on the sequence effect from the neighboring 5′ and 3′ flanking bases and a site-specifically incorporated εA lesion across all 16 possible sequence contexts and tested their repair efficiency by the three AlkB family enzymes. The results demonstrated that the repair of εA by the AlkB proteins is sequence-dependent, enzyme-dependent, and strand-dependent (ss-DNA vs. ds-DNA). Whereas ALKBH2 repair of εA was more efficient in ds-DNA, AlkB repair was more efficient in ss-DNA (Table S1). Efficient repair of εA by ALKBH3 only occurred in ss-DNA; this finding held true even if a large quantity of ALKBH3 was used for ds-DNA. Consistent with previous work on εA, these findings demonstrate the strand preferences in εA repair across the AlkB family enzymes [12,13,14,16]. Collectively, this study demonstrates the utility of applying for a bottom-up approach to evaluate the repair aspect of mutational spectra. This method holds translational promises for cancer prognosis and diagnosis. The methods developed here can be applied broadly to investigate the mutational patterns of endogenous and exogenous chemicals, including information generated from the formation, replication bypass, and repair steps. Future work is needed to investigate the other two aspects of the εA adduct as well as the repair of εA by other enzymes, such as alkyladenine DNA glycosylase in the base excision repair pathway.

## Supplementary Materials

The following are available online, Experimental Procedures, Figure S1: High resolution ESI-TOF MS analysis of 10mer containing starting material (TXG, GXA) and product (TAG, GAA) after ALKBH2 repair (X = εA), Figure S2: εA repair efficiencies under 16 different sequence contexts (5’ base fixed), Table S1: Repair efficiencies (mean ± SD) of the 16 ss- or ds-DNA sequences containing εA repaired by ALKBH2, ALKBH3 and AlkB.

## Sample Availability

Samples of the oligonucleotides and proteins are available from the authors.

## Abbreviations

εA	1,*N*^6^ ethenoadenine
ROS	reactive oxygen species
UV	ultraviolet
LPO	lipid peroxidation
VC	vinyl chloride
CAA	chloroacetaldehyde
2OG-2	2-oxoglutarate
ss-DNA	single-strand-DNA
ds-DNA	double-strand-DNA
*m*/*z*	mass/charge
Mag	methyladenine DNA glycosylase
hOGG1	human 8-oxoguanine glycosylase 1
