# Supplementary material for: Sequence Dependent Repair of 1,N6-Ethenoadenine by DNA Repair Enzymes ALKBH2, ALKBH3, and AlkB
Source: Molecules. 2021 Aug 31;26(17):5285. doi: 10.3390/molecules26175285 (PMC8434105; doi:10.3390/molecules26175285)
Supplement: Supplementary file 1 [file molecules-26-05285-s001.zip › molecules-1332548-supplementary.pdf]

Sequence dependent repair of 1,N^6-ethenoadenine by DNA repair enzymes ALKBH2, ALKBH3 and AlkB

Rui Qi†, Ke Bian†, Fangyi Chen†, Qi Tang†, Xianhao Zhou† and Deyu Li†,*

†Department of Biomedical and Pharmaceutical Sciences, College of Pharmacy, University of Rhode Island, Kingston, Rhode Island 02881, United States

Corresponding Author

*E-mail: deyuli@uri.edu

TABLE OF CONTENTS

EXPERIMENTAL PROCEDURES

Figure S1. High resolution ESI-TOF MS analysis of 10mer containing starting material (TXG, GXA) and product (TAG, GAA) after ALKBH2 repair (X= εA).

Figure S2. εA repair efficiencies under 16 different sequence contexts (5' base fixed).

Table S1. Repair efficiencies (mean ± SD) of the 16 ss- or ds-DNA sequences containing εA repaired by ALKBH2, ALKBH3 and AlkB.

REFERENCES

EXPERIMENTAL PROCEDURES

Synthesis of Oligonucleotides Containing εA. Sixteen 10mer oligonucleotides with the sequence 5′-GACCNXNGCC-3′ (X= εA, N=A/C/G/T) were made by using a solid-phase phosphoramidite method on a MerMade-4 Oligonucleotide synthesizer.[1,2] The phosphoramidites were purchased from ChemGenes. The final cleavage and deprotection step were carried out by treating the oligonucleotide with concentrated aqueous ammonium hydroxide (30%) at 25 °C for 36 h. Oligonucleotide concentrations were determined by measuring UV absorbance at 260 nm. The extinction coefficient (ε) of εA adduct is calculated as unmodified A because of the negligible difference between the values in the context of a 10mer DNA oligonucleotide.

Purification of εA Containing Oligonucleotides by HPLC. Newly synthesized oligonucleotides were purified by reverse-phase (C18) HPLC method.[1] The purifications were performed by using a Phenomenex Luna C18 column (4.60 × 150 mm, 5 μm) at a flow rate of 1.7 mL/min. Solvent A was 100 mM triethylammonium acetate in water, and solvent B was 100% acetonitrile. The solvent gradient was carried out under the following conditions: 4.0% of B for 0.5 min, 4.0 to 7.7% of B over 0.5 min, 7.7 to 9.2% of B over 110 min, 9.2 to 80.0% of B over 1 min, 80.0% of B for 5 min, 80.0 to 4.0% of B over 1 min, and 4.0% B for 5 min. Column oven was set at 25 °C. The UV signal at 260 nm was used to detect the oligonucleotide species.

Expression and Purification of the AlkB Family Proteins. AlkB and its human homologs ALKBH2 and ALKBH3 were expressed and purified as described previously.[2,3] In brief, his-tagged AlkB was obtained by transforming pET28a(+)-AlkB into Rosetta2(DE3)pLysS *E. coli* cell (BL21(DE3)pLysS *E. coli* cell for ALKBH2 and ALKBH3), and protein expression was induced by the addition of 1 mM isopropyl β-D-thiogalactopyranoside (IPTG) at 37 °C (37 °C for ALKBH2 and 30 °C for ALKBH3). The his-tagged proteins were purified by affinity chromatography with HisTrap HP (GE Healthcare Life Sciences). Thrombin (Sigma-Aldrich, 0.005 U/10μg protein) was used to digest his-tag containing protein overnight. The final purified protein was stored at −80 °C in the AlkB protein buffer as previously described.[2]

***In vitro* Enzymatic Reactions.** The enzymatic reactions were conducted in order to compare the εA repair efficiencies under 16 different sequence contexts. Both ss- and ds-DNA substrates were used for these repair reactions. The enzymatic reactions utilizing ss-DNA were incubated at 37 °C for 1 h in the presence of necessary cofactors in a 20 μL reaction volume [70.0 μM $Fe(NH_4)_2(SO_4)_2 \cdot 6H_2O$, 0.93 mM α-KG, 1.86 mM ascorbic acid, and 46.5 mM HEPES (pH 8.0)]. The reactions were quenched by adding 10.0 mM EDTA followed by heating up to 95 °C for 5 min. For the ds-DNA substrates, 1.25 equiv. of the 16mer complementary oligonucleotides, 5′-GCATGCGGCNTNGGTC-3′ (N=G/A/T/C), were annealed with 10mer εA containing oligonucleotides by heating the mixture at 95 °C for 5 min and then gradually cooling down to room temperature at a rate of 1 °C/ min. Reaction temperature was set to 25 °C, which is lower than the melting temperature of ds-DNA. The ds-DNA reactions were quenched by adding 10.0 mM EDTA together with 1.5 equiv. of 16mer oligonucleotides, 5′- GACCNANGCCGCATGC -3′ (N=G/A/T/C, to form a new ds-DNA with the1.25 equiv. 16mer complementary strand added previously and release the 10 mer starting material and product), heated up to 95 °C for 5 min, and cooled down to room temperature at a rate of 1 °C/ min.[4] Each reaction was performed in triplicate.

LC-MS Analysis. Analyses of oligonucleotides were performed by LC-ESI-TOF-MS (AB Sciex, ABI4600), and all data represent the mean ± standard deviation (SD) of three independent experiments. The heating process after the reaction greatly decreased the levels of reaction intermediates (epoxide and glycol in Figure 1 in the main manuscript). So, the repair efficiency of εA was obtained by calculating the ratio of peak area of product Adenine (A) to the total amount of starting material and product (εA + A). ESI was conducted by using a needle voltage of 4.0 kV in a negative ion mode. A heated capillary was set at 300 °C. The nebulizer gas pressure was 30 psi; the heater gas pressure was 70 psi; the curtain gas pressure was 30 psi; the declustering potential was −200 V; and the collision energy was -5 V. Liquid chromatographic separation was achieved by using an Acclaim Polar Advantage II C18 column (2.1 × 250 mm; 3 μm) at a flow rate of 0.1 mL/min. Solvent A was 500 mM 1,1,1,3,3,3-hexafluoro-2-propanol (HFIP) in water, and solvent B was 500 mM HFIP in 50% methanol. A solvent gradient was carried out under the following conditions: 30% of B for 3 min, 30 to 51% of B over 42

min, 51 to 70% of B over 1 min, 70% of B for 6 min, 70 to 30% of B over 1 min, and 30% B over 12 min. LC column oven was set at 35 °C during whole running time.

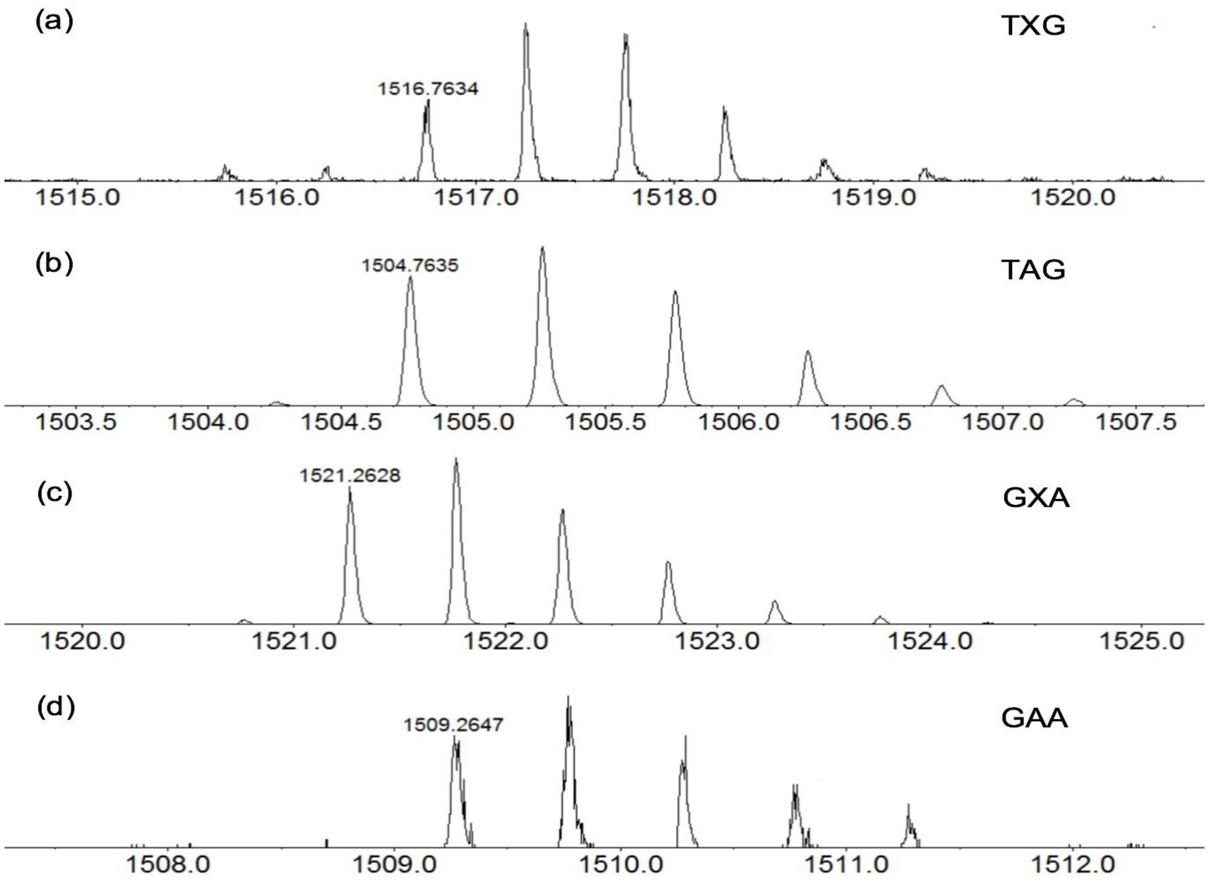

Figure S1. High resolution ESI-TOF MS analysis of 10mer containing starting material (TXG, GXA) and product (TAG, GAA) after ALKBH2 repair (X= εA). TXG and GXA are selected since they showed the highest and lowest repair respectively among 16 sequence contexts. Data represent the −2 charge envelope, and the monoisotopic peak (all 12C, 14N, etc.) values are labeled above the first peak in each peak envelope. (a) TXG (b) TAG; (c) GXA; and (d) GAA.

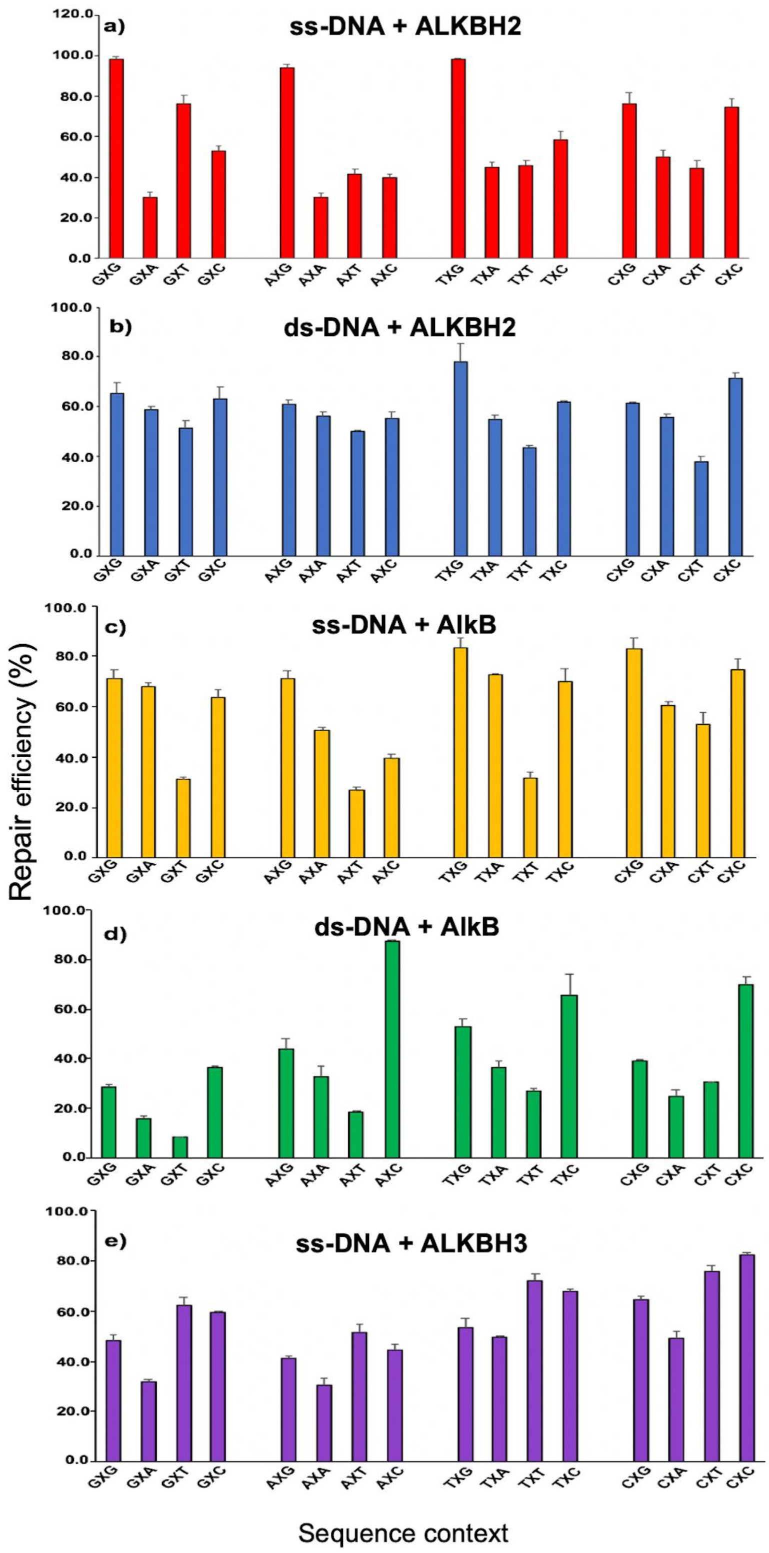

Figure S2. εA repair efficiencies under 16 different sequence contexts (5' base fixed). The oligonucleotide concentration was fixed at 2.5μM. Distinct conditions are shown as follows: **a)** ss-DNA+2.2μM ALKBH2 (red); **b)** ds-DNA+1.1μM ALKBH2 (blue); **c)** ss-DNA+0.4μM AlkB (yellow); **d)** ds-DNA+1.1μM AlkB (green); and **e)** ss-DNA+4μM ALKBH3 (purple). Percentage of repair for all reactions is summarized in Table S1. Error bars represent the standard deviation from triplicate experiments.

Table S1. Repair efficiencies (mean ± SD) of the 16 ss- or ds-DNA sequences containing εA repaired by ALKBH2, ALKBH3 and AlkB. For oligonucleotide synthesis, the sequence of the 10mer was 5′-GACCNXNGCC -3′, where X = εA and N = G/A/T/C.

	2.5μM ss-εA+2.2μM ALKBH2	2.5μM ds-εA+1.1μM ALKBH2	2.5μM ss-εA+0.4μM AlkB	2.5μM ds-εA+1.1μM AlkB	2.5μM ss-εA+4.0μM ALKBH3
AXA	30.2±1.9	56.1±1.8	50.4±1.5	32.4±4.7	30.7±2.7
CXA	50.1±3.2	55.9±1.1	60.4±1.5	24.9±2.7	49.3±2.5
GXA	30.0±2.5	59.1±1.2	68.0±1.4	15.5±1.0	32.0±1.0
TXA	45.1±2.6	54.9±1.9	72.4±0.7	36.6±2.2	49.6±0.6
AXC	39.8±1.8	55.4±2.6	39.6±1.3	87.4±0.5	44.5±2.1
CXC	74.8±4.0	71.3±2.1	74.6±4.4	69.7±3.5	82.3±1.1
GXC	53.1±2.4	63.0±4.8	63.6±3.1	36.4±0.4	59.6±0.1
TXC	58.3±4.3	62.1±0.2	69.7±5.4	65.8±8.5	67.9±0.6
AXG	94.0±1.7	61.3±1.5	70.9±3.2	44.0±4.1	41.2±1.1
CXG	76.0±5.8	61.3±0.5	82.7±4.4	38.8±0.9	64.6±1.1
GXG	98.1±1.2	65.2±4.5	71.1±3.6	28.6±1.0	48.1±2.2
TXG	98.1±0.4	77.9±7.3	83.1±4.3	52.7±3.4	53.3±3.6
AXT	41.5±2.7	50.3±0.2	27.0±1.2	18.5± 0.2	51.7±2.9
CXT	44.4±3.8	37.9±2.1	52.8±5.0	30.3±0.1	75.8±2.5
GXT	76.4±3.9	51.6±3.0	31.3±0.9	8.3±0.2	62.0±3.6
TXT	45.7±2.4	43.6±0.8	31.8±2.3	26.9±0.9	72.1±2.9

References

1. Tang, Q. *et al.* Characterization of Byproducts from Chemical Syntheses of Oligonucleotides Containing 1-Methyladenine and 3-Methylcytosine. *ACS Omega* **2**, 8205–8212 (2017).
2. Chen, F. *et al.* Adaptive Response Enzyme AlkB Preferentially Repairs 1-Methylguanine and 3-Methylthymine Adducts in Double-Stranded DNA. *Chem. Res. Toxicol.* **29**, 687–693 (2016).
3. Fu, D., Samson, L. D., Hübscher, U. & van Loon, B. The interaction between ALKBH2 DNA repair enzyme and PCNA is direct, mediated by the hydrophobic pocket of PCNA and perturbed in naturally-occurring ALKBH2 variants. *DNA Repair* **35**, 13–18 (2015).
4. Chen, F. *et al.* Oncometabolites d- and l-2-Hydroxyglutarate Inhibit the AlkB Family DNA Repair Enzymes under Physiological Conditions. *Chem. Res. Toxicol.* **30**, 1102–1110 (2017).
